# From the West Suffolk General Hospital, Bury St. Edmund's

**Published:** 1920-02-21

**Authors:** Eva E. Hardwicke

**Affiliations:** Secretary.


**From the West Suffolk General Hospital, Bury St. Edmund's.**

*To the Editor of* The Hospital.

Sir,—As you kindly gave me a word of commendation for the early publication of my Hospital Report in which you mentioned the fact that the accounts were not published on the Burdett system, I thought you might like to know that 1919 is the last year that this will be the case, as my Committee have now given me permission to work on the approved system. I hope, therefore, that the 1920 Report will show all the figures on the Uniform System. I do not know if it will be as early, as I was rather worn out after this year's effort.—Yours faithfully,

Eva E. Hardwicke, *Secretary*.

